# Association of a Single Nucleotide Variant in *TERT* with Airway Disease in Japanese Rheumatoid Arthritis Patients

**DOI:** 10.3390/genes14112084

**Published:** 2023-11-16

**Authors:** Takashi Higuchi, Shomi Oka, Hiroshi Furukawa, Kota Shimada, Shinichiro Tsunoda, Satoshi Ito, Akira Okamoto, Misuzu Fujimori, Tadashi Nakamura, Masao Katayama, Koichiro Saisho, Satoshi Shinohara, Toshihiro Matsui, Kiyoshi Migita, Shouhei Nagaoka, Shigeto Tohma

**Affiliations:** 1 Department of Clinical Research, NHO Tokyo National Hospital, 3-1-1 Takeoka, Kiyose 204-8585, Japan; takashi.qef@ac.auone-net.jp (T.H.); oka-tkb@umin.org (S.O.); touma.shigeto.jy@mail.hosp.go.jp (S.T.); 2 Clinical Research Center for Allergy and Rheumatology, NHO Sagamihara National Hospital, 18-1 Sakuradai, Minami-ku, Sagamihara 252-0392, Japan; ninja02matsui@gmail.com; 3 Department of Rheumatology, NHO Sagamihara National Hospital, 18-1 Sakuradai, Minami-ku, Sagamihara 252-0392, Japan; kouta_shimada@tmhp.jp; 4 Department of Rheumatic Diseases, Tokyo Metropolitan Tama Medical Center, 2-8-29 Musashi-dai, Fuchu 183-8524, Japan; 5 Division of Rheumatology, Department of Internal Medicine, Hyogo College of Medicine, 1-1 Mukogawa-cho, Nishinomiya 663-8501, Japan; tsunoda-shinichi@sumitomo-hp.or.jp; 6 Department of Nephrology, Sumitomo Hospital, 5-3-20 Nakanoshima, Kita-ku, Osaka 530-0005, Japan; 7 Department of Rheumatology, Niigata Rheumatic Center, 1-2-8 Hon-cho, Shibata 957-0054, Japan; s-ito@water.ocn.ne.jp; 8 Department of Rheumatology, NHO Himeji Medical Center, 68 Hon-machi, Himeji 670-8520, Japan; cag93700@pop13.odn.ne.jp (A.O.);; 9 Department of Rheumatology, Sakurajyuji Hospital, 1-1-1 Miyukikibe, Minami-ku, Kumamoto 861-4173, Japan; t1.nakamura@sakurajyuji.jp; 10 Department of Internal Medicine, NHO Nagoya Medical Center, 4-1-1 Sannomaru, Naka-ku, Nagoya 460-0001, Japan; katayama.masao.ec@mail.hosp.go.jp; 11 Department of Orthopedics/Rheumatology, NHO Miyakonojo Medical Center, 5033-1 Iwayoshi-cho, Miyakonojo 885-0014, Japan; saisho889@miyazaki-catv.ne.jp; 12 Tanimura Hospital, 10-2 Kitakoji, Nobeoka 882-0041, Japan; 13 Tochigi Rheumatology Clinic, 1-1-9 Ekimaedori, Utsunomiya 321-0964, Japan; shinohara-trc@nifty.com; 14 Clinical Research Center, NHO Nagasaki Medical Center, 2-1001-1 Kubara, Omura 856-8562, Japan; migita@fmu.ac.jp; 15 Department of Gastroenterology and Rheumatology, Fukushima Medical University School of Medicine, 1 Hikarigaoka, Fukushima 960-1295, Japan; 16 Department of Rheumatology, Yokohama Minami Kyosai Hospital, 1-21-1 Rokuura-higashi, Kanazawa-ku, Yokohama 236-0037, Japan; nagaokascrt@msn.com

**Keywords:** single nucleotide variant, airway disease, nonspecific interstitial pneumonia, rheumatoid arthritis, genetic association

## Abstract

Interstitial lung disease and airway disease (AD) are often complicated with rheumatoid arthritis (RA) and have a poor prognosis. Several studies reported genetic associations with interstitial lung disease in RA. However, few genetic studies have examined the susceptibility to AD in RA patients. Here, we investigated whether single nucleotide variants susceptible to idiopathic pulmonary fibrosis might be associated with interstitial lung disease or AD in Japanese RA patients. Genotyping of rs2736100 [C/A] in *TERT* and rs1278769 [G/A] in *ATP11A* was conducted in 98 RA patients with usual interstitial pneumonia, 120 with nonspecific interstitial pneumonia (NSIP), 227 with AD, and 422 without chronic lung disease using TaqMan assays. An association with AD in RA was found for rs2736100 (*p* = 0.0043, Pc = 0.0129, odds ratio [OR] 1.40, 95% confidence interval [CI] 1.11–1.77). *ATP11A* rs1278769 was significantly associated with NSIP in older RA patients (>65 years, *p* = 0.0010, OR 2.15, 95% CI 1.35–3.40). This study first reported an association of rs2736100 with AD in RA patients and *ATP11A* rs1278769 with NSIP in older RA patients.

## 1. Introduction

Rheumatoid arthritis (RA) is a chronic inflammatory disease that destroys synovial joints [1]. RA is often associated with extra-articular manifestations that include interstitial lung disease, airway disease (AD), vasculitis, pleuritis, pericarditis, and Felty’s syndrome [2]. Interstitial lung disease is characterized by interstitial inflammation of the lung and is found in about 10% of RA patients [3]. It confers poor outcomes in RA patients [4]. Interstitial lung disease in RA includes usual interstitial pneumonia (UIP) and nonspecific interstitial pneumonia (NSIP); RA patients with UIP are associated with a poor prognosis [5]. AD is also frequently detected in RA [6,7,8,9] and worsens its prognosis [10,11]. Thus, it is necessary to clarify the pathogenesis of interstitial lung disease and AD in RA.

The pathogenesis of RA is still unknown, but the susceptibility is thought to be influenced by genetic and environmental factors. In our previous study, the *MUC5B* single nucleotide variant (SNV) was revealed to be associated with RA-associated interstitial lung disease [12]. The *MUC5B* gene encodes mucin 5B, a secretory mucin expressed in the lung. The risk allele of the SNV upregulates mucin 5B expression and might interfere alveolar repair. In a study by another group, an SNV in *RPA3-UMAD1* was also associated with RA-associated interstitial lung disease [13], but this was not confirmed in our replication study [14]. Associations of *TOLLIP*, *MUC5B*, *TERT*, and *FAM13A* SNVs with interstitial lung disease were reported in RA patients from European populations [15]. *TOLLIP* encodes an adaptor protein that regulates the degradation of the TGF-β type 1 receptor. The SNV in *FAM13A* was confirmed to be associated with interstitial lung disease in our study [16]. The FAM13A protein plays some roles in the Wnt signaling pathway involved in the pathogenesis of idiopathic pulmonary fibrosis. Taken together, these studies have demonstrated genetic associations with interstitial lung disease in RA. In contrast, few genetic studies have investigated the susceptibility to AD in RA [7,17,18,19,20,21].

Associations of *TERT* or *ATP11A* with idiopathic pulmonary fibrosis were reported in previous studies [22,23,24]. *TERT* was also associated with interstitial lung disease in RA [15] and combined pulmonary fibrosis and emphysema [25]. *TERT* encodes a component of the telomerase complex that regulates the telomere length and cell survival. *ATP11A* is expressed in the lung and the methylation of *ATP11A* correlated with the severity of cystic fibrosis [26]. *TERT* and *ATP11A* might be candidate causative genes for interstitial lung disease or AD in RA. This study was performed to clarify whether SNVs in *TERT* and *ATP11A* are associated with interstitial lung disease or AD in Japanese RA patients.

## 2. Materials and Methods

### 2.1. Patients

RA patients fulfilled the Rheumatoid Arthritis Classification Criteria [27] or American College of Rheumatology Criteria for RA [28] and were native Japanese and living in Japan. RA patients with chest conventional or high-resolution computed tomography (CT) images as clinical information were recruited from 2010 to 2023 at the hospitals of the research group organized by NHO Tokyo National Hospital. Based on the predominant findings of CT images interpreted by two rheumatologists, specialists of lung diseases in RA [21], RA patients were diagnosed with UIP, NSIP, AD, or no chronic lung disease (CLD). Patients with other predominant findings of CT were excluded.

*TERT* rs2736100 and *ATP11A* rs1278769 allele frequencies in the Japanese population were obtained from the 38KJPN Japanese Multi Omics Reference Panel (https://jmorp.megabank.tohoku.ac.jp/202206/, accessed on 19 April 2022) [29].

This study was conducted in accordance with the Declaration of Helsinki and was approved by the Tokyo National Hospital Research Ethics Committee (190010) and Research Ethics Committees of all other institutes involved in this study. In this study, written informed consent was obtained from each patient.

### 2.2. Genotyping

Genomic DNA was extracted from peripheral blood of the RA patients. Genotyping of rs2736100 [C/A] in *TERT* and rs1278769 [G/A] in *ATP11A* was conducted from the genomic DNA of RA patients with TaqMan assays (Assay ID: C___1844009_10 and C___8701049_10, respectively, Thermo Fisher Scientific Inc., Waltham, MA, USA) using Real-Time PCR System.

### 2.3. Statistical Analysis

BellCurve for Excel (Social Survey Research Information, Tokyo, Japan) was employed for statistical analyses. Clinical manifestations of RA patients were analyzed using Mann–Whitney U test or Fisher’s exact test using 2 × 2 contingency tables. Associations of the SNVs were analyzed using Fisher’s exact test or Cochran-Armitage test. For the adjustment of multiple comparisons, the Bonferroni method was used: *p* values were multiplied by the number of tests to generate corrected *p* values (Pc). *p* values less than 0.05 were considered to indicate statistical significance. Statistical power of 80% was obtained when the odds ratio (OR) was 1.40 (AD vs. CLD(-)) or higher for rs2736100. It was calculated to be 1.63 (NSIP vs. CLD(-)) for rs1278769 (http://biostat.mc.vanderbilt.edu/wiki/Main/PowerSampleSize, accessed on 19 April 2022) [30].

## 3. Results

### Association of TERT rs2736100 with AD in RA and ATP11A rs1278769 with NSIP in RA

A total of 98 RA patients with UIP, 120 with NSIP, 227 with AD, and 422 without CLD were recruited to the study (Table 1). TERT rs2736100 and ATP11A rs1278769 were genotyped in the RA patients (Table 2). A deviation from the Hardy–Weinberg equilibrium was not observed for TERT rs2736100 (*p* = 0.3019), but it was observed in ATP11A rs1278769 (*p* = 0.0110). We investigated whether TERT rs2736100 was associated with AD, UIP, or NSIP in Japanese RA patients (Table 2, Supplementary Table S1). TERT rs2736100 was associated with AD under the allele (*p* = 0.0043, Pc = 0.0129, OR 1.40, 95% confidence interval [CI] 1.11–1.77), recessive (*p* = 0.0074, Pc = 0.0222, OR 1.84, 95% CI 1.18–2.86), and codominant models (*p* = 0.0042, Pc = 0.0127). No association with UIP or NSIP in RA was detected for TERT rs2736100. No association with UIP or AD in RA was detected for ATP11A rs1278769.

To exclude the effects of age differences between the AD and CLD(-) groups, the association of TERT rs2736100 was investigated in older RA patients (>65 years, Table 3). TERT rs2736100 was still significantly associated with AD in older RA patients (*p* = 0.0438, OR 1.40, 95% CI 1.02–1.92). Analogically, the association of ATP11A rs1278769 was also investigated in older RA patients because age differences were detected between the NSIP and CLD(-) groups. An association of ATP11A rs1278769 with NSIP in older RA patients was found (*p* = 0.0010, OR 2.15, 95% CI 1.35–3.40). Thus, the association of TERT rs2736100 with AD was found in Japanese RA patients and ATP11A rs1278769 was significantly associated with NSIP in older RA patients.

We also investigated whether TERT rs2736100 or ATP11A rs1278769 were associated with RA per se (Table 4). However, these variants were not associated with RA.

## 4. Discussion

We detected that *TERT* rs2736100C is predisposing to AD in RA patients in Japan. An association of *TERT* rs2736100 with interstitial lung disease was known in European RA patients [15], but this is the first study, to the best of our knowledge, to reveal an association of the SNV with AD. *ATP11A* rs1278769 was also found to be associated with NSIP in older RA patients. *ATP11A* was previously found to be associated with idiopathic pulmonary fibrosis [22,23], but the association of this SNV with NSIP in RA has not been reported.

*TERT* rs2736100A was reported to be a risk allele for idiopathic pulmonary fibrosis [22,23,24] and interstitial lung disease in European RA patients [15]. *TERT* rs7726159 was previously investigated in Mexican and European populations [12], but it was not associated with interstitial lung disease in RA. This allele was associated with lower levels of *TERT* gene expression [31] and with a shorter leukocyte telomere length [32], which contributed to the development of idiopathic pulmonary fibrosis [33]. However, in this study, *TERT* rs2736100C, a protective allele for idiopathic pulmonary fibrosis, was found to be a risk allele for AD in RA. These results suggested that a high *TERT* gene expression and long leukocyte telomere length might contribute to the development of AD in RA, although dysfunction of the telomere was involved in the development of AD [34]. *TERT* rs2736100C was also associated with combined pulmonary fibrosis and emphysema [25]. *TERT* rs7726159A was a risk allele for systemic lupus erythematosus in Asian populations [35] and was in moderate linkage disequilibrium with rs2736100C in Japanese populations (D′ = 0.932, r2 = 0.768, http://www.ensembl.org/, accessed on 16 April 2022). Moreover, *TERT* rs2736100A was a risk allele for microscopic polyangiitis [36]. These data suggest that SNVs in *TERT* might be involved in the development of autoimmune features in a protective or predisposing manner.

It was reported that *ATP11A* was associated with idiopathic pulmonary fibrosis [22,23]. *ATP11A* rs1278769 was previously tested in Mexican and European and populations [12], but it was not associated with interstitial lung disease in RA. The *ATP11A* gene was reported to be expressed in the lung and its methylation correlated to the severity of cystic fibrosis [26]. In the present study, *ATP11A* rs1278769G was revealed to be associated with NSIP in older RA patients. The Genotype-Tissue Expression (GTEx) database showed an association of rs1278769G with the down-regulation of *ATP11A* in the lung (*p* = 5.0 × 10^−11^, Supplementary Figure S1, https://gtexportal.org/home/snp/rs1278769, accessed on 17 April 2022) [37]. Low expression levels of *ATP11A* were associated with the severity of Coronavirus Disease 2019 [38]. In our study, *ATP11A* rs1278769 was associated with NSIP in older RA patients, but it was not associated with UIP in RA patients, suggesting the heterogeneity of the pathogenesis of RA chronic lung disease.

To the best of our knowledge, the present study is the first report on an association of *TERT* rs2736100 with AD in RA and *ATP11A* rs1278769 with NSIP in older RA patients. This study included several limitations: the sample size was modest, and it was based on the results of Japanese populations. Multi-ethnic and larger-scale studies should be conducted to evaluate the etiology of AD or NSIP in RA.

## 5. Conclusions

In conclusion, this study showed associations of *TERT* rs2736100 with AD in RA and *ATP11A* rs1278769 with NSIP in older RA patients, suggesting the heterogeneous pathogenesis of chronic lung disease in RA patients.

## Figures and Tables

**Table 1:** Characteristics of the RA patients.

	UIP	NSIP	AD	CLD(-)
Number	98	120	227	422
Male, *n* (%)	44 (44.9)	41 (34.2)	42 (18.7)	66 (15.7)
*p* values	* 2.63 × 10^−9^	* 2.42 × 10^−5^	* 0.3762	
Mean age, years (SD)	71.3 (9.9)	68.0 (10.4)	67.1 (11.6)	61.6 (12.7)
*p* values	9.78 × 10^−13^	2.01 × 10^−7^	6.44 × 10^−9^	

RA: rheumatoid arthritis; UIP: usual interstitial pneumonia; NSIP: nonspecific interstitial pneumonia; AD: airway disease; CLD: chronic lung disease. Percentages or standard deviations are shown in parentheses; SD: standard deviation. RA patients with chest conventional or high-resolution computed tomography images were included; RA patients with diagnoses other than UIP, NSIP, AD, or no CLD were excluded. Statistical differences were compared with CLD(-) group using Mann–Whitney U test or Fisher’s exact test using 2 × 2 contingency tables. * Fisher’s exact test was used.

**Table 2:** Genotype frequencies of TERT rs2736100 and ATP11A rs1278769 in the RA patients.

*TERT*		Genotype			Allele	Allele model			
rs2736100	*n*	[C/C]	[C/A]	[A/A]	[C]	*p*	OR	95% CI	*P*c
UIP(+)RA, n (%)	98	12 (12.2)	40 (40.8)	46 (46.9)	64 (32.7)	0.2836	0.83	(0.59–1.15)	0.8508
NSIP(+)RA, n (%)	120	16 (13.3)	59 (49.2)	45 (37.5)	91 (37.9)	0.8205	1.04	(0.77–1.40)	NS
AD(+)RA, n (%)	227	45 (19.8)	115 (50.7)	67 (29.5)	205 (45.2)	0.0043	1.40	(1.11–1.77)	0.0129
CLD(-)RA, n (%)	422	50 (11.8)	212 (50.2)	160 (37.9)	312 (37.0)				
*ATP11A*		Genotype			Allele	Allele model			
rs1278769	*n*	[G/G]	[G/A]	[A/A]	[G]	*p*	OR	95% CI	*P*c
UIP(+)RA, n (%)	98	45 (45.9)	42 (42.9)	11 (11.2)	132 (67.3)	0.6070	0.91	(0.65–1.27)	NS
NSIP(+)RA, n (%)	120	75 (62.5)	33 (27.5)	12 (10.0)	183 (76.3)	0.0439	1.41	(1.01–1.97)	0.1317
AD(+)RA, n (%)	227	117 (51.5)	94 (41.4)	16 (7.0)	328 (72.2)	0.3078	1.15	(0.89–1.47)	0.9234
CLD(-)RA, n (%)	422	214 (50.7)	158 (37.4)	50 (11.8)	586 (69.4)				

RA: rheumatoid arthritis; UIP: usual interstitial pneumonia; NSIP: nonspecific interstitial pneumonia; AD: airway disease; CLD: chronic lung disease; OR: odds ratio; CI: confidence interval. Genotype and allele frequencies are shown in parentheses (%). Associations were tested using Fisher’s exact test using 2 × 2 contingency tables under the allele model.

**Table 3:** Genotype frequencies of *TERT* rs2736100 and *ATP11A* rs1278769 in RA patients older than 65 years.

*TERT*		Genotype			Allele	Allele model		
Age > 65	*n*	[C/C]	[C/A]	[A/A]	[C]	*p*	OR	95% CI
AD(+)RA, n (%)	137	23 (16.8)	78 (56.9)	36 (26.3)	124 (45.3)	0.0438	1.40	(1.02–1.92)
CLD(-)RA, n (%)	191	26 (13.6)	90 (47.1)	75 (39.3)	142 (37.2)			
*ATP11A*		Genotype			Allele	Allele model		
Age > 65	*n*	[G/G]	[G/A]	[A/A]	[G]	*p*	OR	95% CI
NSIP(+)RA, n (%)	78	53 (67.9)	22 (28.2)	3 (3.8)	128 (82.1)	0.0010	2.15	(1.35–3.40)
CLD(-)RA, n (%)	191	95 (49.7)	70 (36.6)	26 (13.6)	260 (68.1)			

RA: rheumatoid arthritis; CLD: chronic lung disease; NSIP: nonspecific interstitial pneumonia; OR: odds ratio; CI: confidence interval; AD: airway disease. Allele and genotype frequencies are shown in parentheses (%). Associations were tested in comparison with the CLD(-) RA older than 65 years using Fisher’s exact test.

**Table 4:** Allele frequencies of *TERT* rs2736100 and *ATP11A* rs1278769 in the RA patients and controls.

*TERT*		Allele	Allele model		
rs2736100	*n*	[C]	*p*	OR	95% CI
RA, n (%)	867	672 (38.8)	0.5027	0.97	(0.88–1.07)
Control, n (%)	38,721	30,635 (39.6)			
*ATP11A*		Allele	Allele model		
rs1278769	*n*	[G]	*p*	OR	95% CI
RA, n (%)	867	1229 (70.9)	0.5953	1.03	(0.93–1.14)
Control, n (%)	38,720	54,405 (70.3)			

RA: rheumatoid arthritis; OR: odds ratio; CI: confidence interval. Allele frequencies are shown in parentheses (%). Association was tested in the comparison with the control population using Fisher’s exact test using 2 × 2 contingency tables.

## Data Availability

Data supporting the findings of this study will be provided upon reasonable request to the authors. However, the clinical information and genotype data of each participant are not available based on the conditions of informed consent and the Act on the Protection of Personal Information.

## Supplementary Materials

The following supporting information can be downloaded at: https://www.mdpi.com/article/10.3390/genes14112084/s1, Figure S1: An association of rs1278769G with low expression levels of the *ATP11A* gene in the lung; Table S1: Associations of rs2736100 *TERT* and rs1278769 *ATP11A* with the RA subsets.
