# Supplementary material for: Association of a Single Nucleotide Variant in TERT with Airway Disease in Japanese Rheumatoid Arthritis Patients
Source: Genes (Basel). 2023 Nov 16;14(11):2084. doi: 10.3390/genes14112084 (PMC10671651; doi:10.3390/genes14112084)
Supplement: Supplementary file 1 [file genes-14-02084-s001.zip › RA-AD Table#4-S1.1.pdf]

Supplementary Table S1. Associations of rs2736100 *TERT* and rs1278769 ATP11A with the RA subsets.

TERT	Dominant model for C				Recessive model for C				Codiminant model	
rs2736100	*P*	OR	95%CI	*P*c	*P*	OR	95%CI	*P*c	*P*	*Pc*
UIP(+)RA, n (%)	0.1091	0.69	(0.44-1.07)	0.3273	0.8641	1.04	(0.53-2.03)	NS	0.1892	0.5675
NSIP(+)RA, n (%)	1.0000	1.02	(0.67-1.55)	NS	0.6378	1.14	(0.63-2.09)	NS	0.8117	NS
AD(+)RA, n (%)	0.0382	1.46	(1.03-2.06)	0.1146	0.0074	1.84	(1.18-2.86)	0.0222	0.0042	0.0127
CLD(-)RA, n (%)										

ATP11A	Dominant model for G				Recessive model for G				Codiminant model	
rs1278769	*P*	OR	95%CI		*P*	OR	95%CI	*P*c	*P*	*Pc*
UIP(+)RA, n (%)	1.0000	1.06	(0.53-2.13)	NS	0.4330	0.83	(0.53-1.28)	NS	0.5035	NS
NSIP(+)RA, n (%)	0.6297	1.21	(0.62-2.35)	NS	0.0229	1.62	(1.07-2.46)	0.0687	0.0347	0.1042
AD(+)RA, n (%)	0.0572	1.77	(0.98-3.19)	0.1716	0.8694	1.03	(0.75-1.43)	NS	0.4682	NS
CLD(-)RA, n (%)										

RA: rheumatoid arthritis, UIP: usual interstitial pneumonia, NSIP: nonspecific interstitial pneu-monia, AD: airway disease, CLD: chronic lung disease, OR: odds ratio, CI: confidence interval. Associations were tested by Fisher's exact test using 2×2 contingency tables for dominant or recessive models or Cochran-Armitage test for codominant model.
