# Supplementary material for: Association of a Single Nucleotide Variant in TERT with Airway Disease in Japanese Rheumatoid Arthritis Patients
Source: Genes (Basel). 2023 Nov 16;14(11):2084. doi: 10.3390/genes14112084 (PMC10671651; doi:10.3390/genes14112084)
Supplement: Supplementary file 1 [file genes-14-02084-s001.zip › RA-AD#4-S1.pdf]

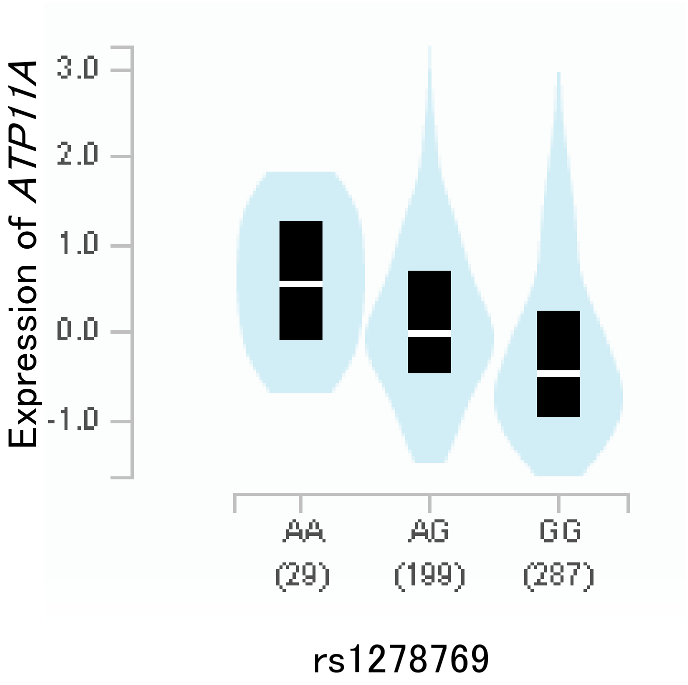

Supplementary Figure S1. An association of rs1278769G with low expression levels of the ATP11A gene in the lung. Expression quantitative trait loci analysis in the Genotype–Tissue Expression portal database (https://gtexportal.org/home/snp/rs1278769) showed an association of rs1278769G with low expression levels of *ATP11A* in the lung (P=5.0 × 10^{-11}).
